# The “floating” valve, a modified bioroot Bentall procedure

**DOI:** 10.3389/fcvm.2026.1760063

**Published:** 2026-03-12

**Authors:** David Derish, Oliver Sebastian Lee, Roupen Hatzakorzian, Dominique Shum-Tim

**Affiliations:** 1 Faculty of Medicine and Health Sciences, McGill University, Montréal, QC, Canada; 2 Faculty of Medicine, Université de Montréal, Montréal, QC, Canada; 3 University College London Medical School, University College London, London, United Kingdom; 4 Department of Anaesthesia, Division of Adult Anaesthesia, McGill University Health Centre, Montréal, QC, Canada; 5 Division of Cardiac Surgery and Surgical Research, McGill University Health Centre, Montréal, QC, Canada

**Keywords:** aortic root replacement, Bentall operation, bioprosthetic valve, prosthesis-patient mismatch, valve degeneration, surgical innovation

## Abstract

With a rising incidence of aortic root disease globally, the Bentall procedure remains the gold standard for aortic root replacement. Unfortunately, the classic mechanical heart valves (MHVs) often employed in these surgeries require lifelong anticoagulation, carrying serious bleeding or thrombo-embolic risks and incurring significant lifestyle changes for patients. Recent advances have shifted focus towards biological heart valves (BHVs), leading to the emergence of a bioroot Bentall which can integrate a “floating” BHV within a synthetic vascular conduit. We reviewed contemporary evidence on this surgery to define indications, outcomes and knowledge gaps. This narrative review highlights surgical techniques, patient selection criteria, and surgical outcomes. Uniquely, “floating” valve placement improves hemodynamics and reduces prosthesis-patient mismatch. Additional advantages included valve-in-valve (ViV) feasibility and easier coronary re-access, while persisting barriers were uncertain long-term BHV durability and a lack of long-term randomized evidence. Emerging technologies, such as advanced biomaterials, and global demand for cardiac surgical care, are likely to further popularise this type of bioroot Bentall. Clinical preferences are shifting towards anticoagulation-free solutions, especially for younger, small-annulus, or anti-coagulation-averse patients. The “floating” bioroot Bentall is a compelling alternative to the use of traditional mechanical prostheses, offering a balance between durability and quality of life. Prospective registries and larger head-to-head trials are now required to benchmark floating bioroot Bentall on survival, valve durability, cost-effectiveness, and patient-reported outcomes. Future research should focus on optimizing BHV durability and refining surgical techniques to further improve clinical outcomes.

## 1 Background

Bentall and DeBono (1) first described their ascending aortic and aortic valve replacement technique in 1968, employing a composite prosthetic graft. Years later, Cabrol documented the excision of small cuffs of aortic tissue around the coronary ostia, modernizing the Bentall procedure with markedly reduced rates of pseudoaneurysm of the aortic and coronary arterial suture lines.

Modern elective Bentall procedures have patient-catered options of stented and stentless valve designs, maximizing structural support, laminar flow, and reduced tissue stress. Mounting evidence supporting the durability and improved hemodynamic performance of BHVs has fuelled their increasing adoption in aortic root replacement. Over time, advancements in BHV design, material engineering, and surgical techniques have improved their durability.

**Figure 1:**
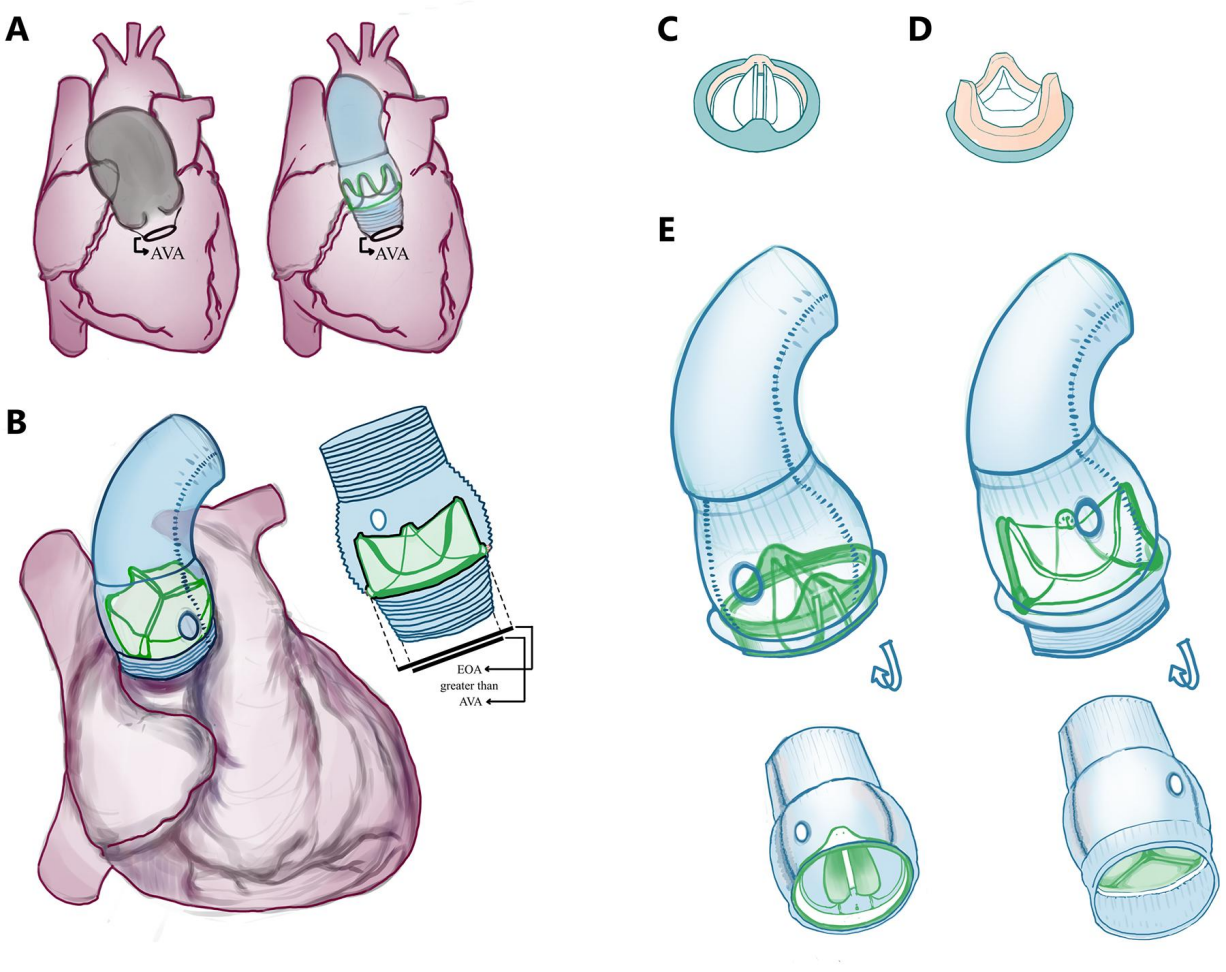
“Floating” valve seating and valve types. **(A)** Diagrammatic representation of the diseased, dilated/aneurysmal aorta on the left, which was repaired via a self-assembled BHV and Dacron Valsalva-type skirted conduit on the right. **(B)** View of valve positioning within the Valsalva skirted conduit, emphasizing the oversizing permitted by the “floating” configuration in the context of a narrow aortic annulus. This figure shows that the valve diameter far exceeds the annular diameter. We highlight how this improves flow dynamics and allows a larger prosthesis in challenging anatomies. It is especially beneficial in patients with a small or frayed annulus, including cases of infective endocarditis, where anastomosis between the valve and the destroyed annular tissue may be challenging. “AVA” = aortic valve annulus; “EOA” = effective orifice area. **(C)** MHV and **(D)** BHV illustrations. **(E)** compares a preassembled composite graft with a mechanical valve versus a skirted self-assembled biologic valve in a Valsalva conduit, emphasizing the skirt and the resulting distance between the annulus and the valve achieved with the “floating” seating.

Importantly, recent paradigm shifts in valve surgery have been driven significantly by patient preferences and improvements in tissue valve technologies. MHVs have historically been prioritized because of their durability and hemodynamics. However, their rigid structure made oversizing impractical, which shaped the longstanding paradigm of valves needing to match the annular size strictly. Unlike MHVs, BHVs are not rigid. As modern techniques emerge and preferences for BHVs grow, valve oversizing is feasible and increasingly common. Procedural innovations allow for reduced cross-clamp times and easier implantation of larger valves. As such, the bioroot Bentall procedure has evolved with a modern approach emphasizing “floating” valve placement (Figure 1), improved procedural efficiency, and future readiness for ViV transcatheter interventions.

## 2 Materials and methods

### 2.1 Review methodology

This review was conducted to analyse shifting paradigms of the bioroot Bentall procedure. A literature search was performed on PubMed. Seminal papers outlining key developments in composite graft technology and self-assembled conduits are highlighted. First, we describe the modified bioroot Bentall procedure via a gross procedural transcript. We then summarize key evidence, showcase advancements, and compare approaches with their alternatives, highlighting indications and limitations. We delve into surgical techniques, then discuss clinical outcomes, reoperation risks, and latest trends.

### 2.2 Surgical technique for this modified “floating” bioroot Bentall

At the McGill University Health Centre (MUHC), we often perform the bioroot Bentall procedure via a self-constructed biologically valved conduit, combining procedural efficiency and improved anatomical versatility. As the procedure begins, a separate sterile table with a secondary team is reserved for conduit construction in parallel. This parallel side-table assembly setup avoids added surgical time; where staffing is limited, one team can assemble the conduit immediately before clamping. Assembly utilises a Dacron Valsalva graft and a bioprosthetic valve 3–5 mm smaller than the graft diameter The Valsalva graft size is selected on the basis of the external diameter of the ascending aorta or proximal arch (typically assessed via preoperative imaging): tube size 24 mm∼BHV size 21 mm; or tube size 30 mm∼BHV 27 mm; tube size ≥32∼BHV 27 mm. This configuration enables supra-annular, skirt-anchored placement of the valve. The annular constraint is thus entirely circumvented. Given universal pre-operative computed tomography (CT) for these patients at the MUHC, CT-derived aortic dimensions inform external root diameters, and intraoperative TEE reliably confirms these. Annular sizers are unnecessary and given the valve's seating within a soft conduit, minor annulus-valve mismatches are well-tolerated. The skirt of the Valsalva graft is everted, and the bioprosthesis is sutured to the base of the sinus segment. Once sewn, the skirt is inverted for a soft inflow segment.

### 2.3 Sizing formulation for the self-assembled “floating” bioroot Bentall

Simultaneously, a median sternotomy exposes the heart, and cardiopulmonary bypass is installed. Arterial cannulation can be established at the ascending aorta for elective cases or at the right subclavian or femoral artery in select cases. The biovalved-conduit is complete before cross-clamp; the aorta is clamped, and a transverse aortotomy is performed above the sinotubular junction. The distal portion of the diseased native aortic root is transected for excision. The aortic valve leaflets are excised, preserving only a small rim of tissue. The annulus can be debrided. Next, the coronary ostia are mobilized by excising “buttons” for later reattachment. Pledgeted horizontal mattress sutures may be placed circumferentially around the annulus, although in some cases, the skirt alone is directly sutured to the native aortic rim via a running suture. Sutures are passed through the lowest portion of the skirt, avoiding the valve cuff itself. The conduit is then lowered into position and secured to the annulus by tying the sutures.

No sizers are necessary. No intraoperative decision on valve size is necessary. Because the valve is not anchored directly into the annulus but rather via the skirt, mismatches between the valve size and annular size are easily tolerated (Figures 1C,D). In patients with annular destruction due to endocarditis or abscess, a pericardial or hemashield patch may be used for annular reconstruction, and the conduit may be sewn in an end-to-end fashion. After the conduit is secured proximally, circular openings in the root graft for new right and left coronary artery ostia are created, and coronary buttons are reimplanted. With the proximal aortic root graft connected to the left ventricular outflow tract, the distal conduit is trimmed and anastomosed to the native aorta or arch (2).

## 3 Discussion

### 3.1 Decision implications

The “classic” Bentall employs a durable mechanical valve prosthesis (Figure 1C) and a rigid sewing ring for structural integrity. The bioroot Bentall with a “floating” valve is a modern modification which incorporates a BHV (Figure 1D) directly in the synthetic conduit, which, at our centre, is assembled intraoperatively often in parallel with the early steps of the procedure. The decision to perform this type of bioroot Bentall aims to minimize cross-clamp time, avoid annular stress, and ease reinterventions. When managing aortopathy, one determines:

1. *Valve-sparing (VSRR) vs. valve replacement (AVR):* Valve-sparing root repair (e.g., David/VSRR) is preferred when cusps are intact, preserving native valve hemodynamics and biocompatibility with improved survival and lower stroke/bleeding risk (3–5). When significant cusp disease or other contraindications exist, a Bentall approach is favoured (3).
2. *MHV vs. BHV:* Mechanical valves offer longevity but require lifelong warfarin (6). Bioprosthetic valves avoid chronic anticoagulation but are prone to structural deterioration and higher reintervention rates in younger patients. The Ross procedure is an alternative for select younger patients with promising long-term results (7–9) yet is generally less suitable in older adults (2, 10). Outcomes across series are mixed and age cut-offs vary (11–17). Contemporary practice trends toward wider BHV use, while comparative studies report heterogeneous results (12, 16–19).
3. *Transcatheter aortic valve implantation (TAVI)* vs. *surgical aortic valve replacement (SAVR) replacement:* TAVI is non-inferior to SAVR across surgical-risk strata in anatomically suitable severe tricuspid AS. This informs the 2025 ESC/EACTS guideline shift toward lifetime-management decision-making (e.g., TAVI favoured ≥70 years; SAVR favored <70 years when low surgical risk). In the modern age, comprehensive decision-making by the Heart Team is emphasized; SAVR permits definitive aortic repair (6, 20, 21).
4. *Commercial vs. self-assembled graft:* Commercial preassembled valve grafts have gained popularity for reducing the operative time, but concerns over bleeding at the proximal anastomosis have emerged (22). These grafts historically contained only MHVs, as BHVs require preservation in solution. With new dry-preservation technologies, BHV-containing conduits are now available at considerable financial cost.

Self-assembled grafts offer technical and financial advantages. They avoid rigid annular sewing and displace hemostatic tension to the pliable graft material. This permits a tension-free anastomosis between hardware (sealed Dacron conduit) and tissue, while the valve prosthesis is tightly sutured to the sewing ring inside the synthetic tube (23). Surgeons may tailor materials to complex anatomy or uniquely damaged tissues. Concerns over operative duration exist, but many reports support negligible increases (2) or no significant impact on outcomes (24). In fact, at our center, the self-assembled valved conduit is constructed before or during patient preparation for cardiopulmonary bypass, avoiding prolongation. Like commercial grafts, outcomes are excellent (23–25), and studies report reduced bypass time, blood loss, and reoperation rates (22). Importantly, self-assembled BHV procedures are more feasible in low-resource settings, where access to commercial BHV conduits remains limited (22, 26–28). Epidemiologically, not only is life expectancy increasing, but the healthcare burden is shifting to noncommunicable diseases, notably cardiovascular disease. The increasing demand for specialized cardiac centres and healthcare infrastructure development call for financially conservative options to manage aortic disease (28).

Table 1 Observational Bentall cohorts show excellent outcomes regardless of prosthesis choice. Mechanical valved conduits minimize valve-related reintervention, whereas biological valved conduits reduce anticoagulation burden. In a large propensity-matched Bentall cohort (*n* = 1,210), operative mortality was low (0.7%) with no early major difference by prosthesis type; however, at 10 years, mortality was higher in the mechanical-conduit group (25.3% vs. 16.4%) while reoperation was higher in the biological-conduit group (7.4% vs. 1.1%). A recent propensity-score matching analysis showed no differences in mean survival time.

**Table 1:** Mechanical vs biological prosthesis in Bentall (valved conduit).

Challenge	MHV	BHV
Anticoagulation	Mandatory warfarin therapy	Only short courses unless otherwise specified
Hemodynamics	Fixed sewing ring and limited upsizing, annulus size constraint more relevant	Enables upsizing with “floating” valve-in-graft, circumventing annular constraint. Gradients may be lower
Redo strategy	Very low valve-related reintervention, but root redo often still requires valve work	Higher late reintervention due to SVD, but modern valve-only redo strategies (i.e., ViV-TAVR) are very well-tolerated
Morbidity	Thromboembolism and bleeding characterise the major long-term risks	SVD is the dominant risk long-term. Lower anticoagulation-related bleeding burden
Patient selection	Very young patient, accepting anticoagulation risks and valve durability	Anticoagulation-averse, small-annulus with risk of PPM, complex annulus
Endocarditis	Catastrophic complication requiring reintervention. No consistent trend in incidence depending on valve type	
Long-term survival	Inconsistent differences for Bentall operations, showing no conclusive superiority/inferiority in valve type across the literature	

SVD: structural valve degeneration; ViV: valve-in-valve; TAVR: transcatheter aortic valve replacement.

Within the specific context of self-assembled biological conduits, several contemporary series emphasize a pragmatic advantage for lifetime management: when degeneration occurs, reintervention may be technically simplified to valve-only replacement within an existing tube (and in selected anatomies, ViV), potentially avoiding redo coronary-button work.

### 3.2 Other relevant techniques

#### 3.2.1 Valve upsizing

Patient-prosthesis mismatch (PPM) occurs when a valve's effective orifice area is insufficient to sustain a patient's cardiac output, leading to outflow obstruction. It results in increased mortality and complications (29). It is more common in younger, female patients with larger body surface areas and coexisting valve disease. PPM is termed nonstructural valve dysfunction, as no apparatus components (i.e., leaflets) are crippled. In contrast, structural valve dysfunction involves leaflet degeneration due to calcification, mechanical stress, or immune rejection (11). To manage PPM, the use of stentless, sutureless, rapid deployment or self-expanding valves enables minimally invasive approaches and reduced bypass/clamp times (30, 31). Although advanced techniques such as the Nicks procedure or annular enlargement offer further solutions, in bioroot Bentall, supraannular valve positioning permits upsizing, constrained only by the patient's outflow tract anatomy.

#### 3.2.2 Reconstruction of frail tissues

Infective endocarditis (IE) involves bloodstream bacterial valve seeding, causing tissue destruction, abscesses, fistulas, vegetation or emboli (2, 32). IE can lead to multi-organ damage. Surgical management requires radical debridement, often causing massive annular fraying. Reconstruction then becomes challenging with a destroyed aortoventricular junction. Surgeons often resort to Bentall variations, such as annular reconstruction using a Hemashield graft. Fragile annuli pose risks: suturing to rigid sewing rings can leave gaps and cause bleeding. Strategies to address annular fragility exist: preservation of native aortic root tissue to reinforce sutures and structural integrity, reinforcement with an additional suture layer to reduce bleeding risk (33), use of a stentless bioconduit (30), or a pericardial skirt for complex reconstructions (34).

#### 3.2.3 *“*Floating” construction

After SAVR with BHVs, PPM remains common (29). While annular or root enlargement can address this, such interventions carry concerning risks (35). An alternative is “floating” valve placement, seating the prosthesis above-annularly. This approach optimizes hemodynamics without adding surgical risk. It is especially indicated for small annuli and patients at high risk of PPM, notably older women (36). In Bentall procedures, it allows for larger prostheses while avoiding oversizing complications, such as AV node stress and heart block (30, 37). “Floating” valves ease future redo operations and are useable in both TAVR and SAVR: valves in similar TAVR show increased EOA and reduced PPM (38, 39). Ongoing studies are examining long-term safety and efficacy in ViV cases (40).

#### 3.2.4 Redo operations

Reintervention is a routine occurrence in BHV implantation because of structural degeneration. ViV TAVR offers a minimally invasive method (41), although its feasibility is limited for valves < 21 mm due to the risks of coronary obstruction, high gradients, and poor survival. The “Matryoshka effect” requires the new valve to be smaller than the previous graft lumen, compounding prosthetic constraints, especially in patients with prior PPM (42, 43).

In degenerated bioroot Bentall cases, reoperation may avoid full root replacement by excising only the degenerated valve. In managing a failed bioroot 11 years postimplantation due to a leaflet tear, one group noted that a retained sewing ring poses sizing challenges for future valves, unless positioned differently (44). Another described valve-on-valve placement in a patient with exceptional annular-coronary distance and limited options (45). Technical challenges of classic aortic root redo include valve excision without disrupting the root replacement and the ensuing placement of new annular sutures. However, in redo operations on a bioroot due to a degenerated valve, full root re-replacement can be avoided by exclusively excising the damaged prosthesis. Broadly, redo root replacements are safe, regardless of dysfunction etiology (degenerative or infectious) (46).

In 2018, Urbanski et al. published their retrospective study on self-assembled Bentall procedures with either MHV or BHV following prior aortic valve or complete root replacement (23). They demonstrated the safety and efficacy of the approach, even with a destroyed annulus. A follow-up paper describing reinterventions on a self-assembled composite BHV reported safety of aortic valve rereplacement in patients with structural valve degeneration (47). Using a floating approach, they achieved strong results in small annuli, with benefits including lower anastomotic tension, minimal bleeding, and simplified reintervention (23).

Urbanski's group also stressed that BHVs do not increase infection nor IE risk. With increasing BHV use, they proposed surgical workarounds for anastomosis in friable annuli after debridement. Typically, surgeons create a tension-free anastomosis for a pliable graft material, then sutured to the sewing ring of a valved conduit (23). Thus, the self-assembled methodology achieves this consistently and safely, without disrupting the implanted coronary buttons, all the while providing surgeons freedom to select materials, valve types, and techniques.

### 3.3 Outcomes and challenges

Bentall complications can occur due to malposition of the perianastomotic suture of the coronary ostia, leading to a variety of poor outcomes, from pseudoaneurysm to dissection (17). Nevertheless, latest evidence suggests that Bentall procedures are efficacious and safe, with an operative risk comparable to that of isolated aortic valve replacement (17). Propensity-adjusted cohorts show similar in-hospital/30-day mortality and major complications for mechanical vs. bioprosthetic composite grafts (12, 19). Self-assembled “floating” bio-root Bentall demonstrates low early mortality and preserved safety even in complex anatomy, including small annuli with supra-annular placement (2, 24, 47). Long-term, adjusted survival is broadly similar between valve types, with mechanical advantage confined to younger patients, while ≥50 years see no survival penalty with BHV (12). Durability, however, diverges as expected: 10-year freedom from reoperation is ∼99% (MHV) vs. ∼93% (BHV) (19). When valve degeneration occurs, valve-only reintervention (surgical valve-in-graft or ViV-TAVR) within the existing conduit avoids redo root/coronary work (19, 25, 41, 43).

Table 2 Across self-assembled bioroot Bentall series, early outcomes are highly dependent on the patient's status and comorbidities: elective sub-groups report very low early mortality, whereas urgent redo/arch-heavy populations carry higher operative risk (49). “Floating” valve-in-graft construction is consistently framed as enabling annulus-independent upsizing and facilitating later intervention. Mid-term survival in large contemporary experience remains acceptable (e.g., 10-year survival ∼70% in a complex 523-patient cohort). Long-term follow-up from a dedicated bio-Bentall program clearly shows that valve degeneration becomes the dominant late issue (50). Thankfully, reintervention can often be limited to the valve within the conduit.

**Table 2:** Early and mid-term outcomes across contemporary self-assembled Bentall/bioroot series.

Study	Construct	Cohort	Early mortality	Mid-term survival	Reintervention
Sirajuddin 2019 (single-centre early experience; *n* = 17)	Valsalva graft + Trifecta; valve sewn into sinus base (skirt-level annular suture)	12 elective, 5 urgent or emergent, 3 redo	0%	Not reported	Not reported
Beckerman 2018 (*n* = 428)	Valsalva graft with freestyle stentless valved conduit	High-risk mix: 31% redo, 59% arch work, 27% CABG, 6% endocarditis	30-day mortality 7% overall; 4% in redo subgroup	Not reported	Valve reintervention 1% for SVD, freedom from AVR was 99% at 6 yrs, 95% at 9 yrs
Patel 2022 (large cohort; *n* = 523)	Valsalva graft with freestyle subcoronary valve sewn	48% urgent, 29% redo, frequent arch work	7.7%	5-yr 83%, 10-yr 71%	Freedom from redo AVR 96% and 99.1% from aortic root redo
Meszaros 2014 (*n* = 201)	Valsalva graft with stented pericardial graft	Mixed pathology: aneurysm 90.5%, acute dissection 5%, endocarditis 4.5%; 12.9% redo	In-hospital 4.5%	1-yr 95.0%, 5-yr 75.2%	Redo 1% (2 patients; endocarditis); freedom from structural valve failure 97.3% at 5y
Stefanelli 2021 (*n* = 30)	Straight Dacron with Medtronic 3F® stentless porcine valve	Root dilatation + AV disease; 6.3% redo; median follow-up of∼7 yrs	0%	10-yr survival 93.1%	Freedom from valve-related reoperation 96.6% at 10y; freedom from SVD 87.5% at 10y
Urbanski 2023/2024 (two-decade update; *n* = 308)	Self-assembled bio-Bentall	Chronic aneurysm predominantly (70%), and remainder with complex root pathology	4.9% overall, 0% in chronic aneurysm subgroup of patients	15-yr 41.9% overall, 62.5% if age <65 yr at time of intervention	15-yr valve reintervention risk 7.7%; SVD management via valve-only redo. Full root redo mainly for acute endocarditis

CABG: coronary artery bypass graft; SVD: structural valve degeneration; AVR: aortic valve replacement; AV: aortic valve.

Similarly, the clinical outcomes of the self-assembled bioroot Bentall procedure are promising at our centre (42), where mean cardiopulmonary bypass time was 169 ± 84 min and aortic cross-clamp time was 110 ± 32 min; operative mortality was 0%; all patients were discharged without long-term anticoagulation. At 6–18 months, left ventricular ejection fraction was 61 ± 6%.

Indirect insights into the bioroot Bentall procedure can be drawn from a comparative study of patients with aortic root dilation undergoing either aortic valve reimplantation (David procedure) or MHV Bentall (4). In patients with healthy native valves, native valve reimplantation yielded superior survival (short- and midterm), despite longer cross-clamp times and greater technical complexity. Poorer outcomes associated with MHV were attributed to thromboembolism, stroke, and endocarditis. We understand this to reflect that the findings are a testament to i. the non-neglieable risks associated with MHVs, and ii. the overwhelming success of The David procedure despite prolonged operative times.

Can the bioroot Bentall offer superior results with its “floating” valve placement? Although bioroot Bentall differs from valve reimplantation, its floating valve design may address many of the same complications. By avoiding rigid intra-annular positioning, this approach potentially mitigates the risks linked to MHV implantation, even in younger patients with dissected aortic roots. Furthermore, in the self-assembled technique, the cross-clamp and bypass times have been shown to remain low (24).

A comparative study evaluated composite vs. self-assembled grafts with cuffs and reported favourable outcomes with the latter (22). While reoperation rates for bioroot Bentall are higher than those for MHV Bentall, this mirrors patterns observed in other BHV procedures. We suspect that as the procedure continues to rise in popularity, its safety and long-term outcomes will develop into a formidable option for a broader category of patients.

### 3.4 Critical appraisal

It is suspected that one reason for the increasing popularity of the BHV is a trend in patient shared decision-making. Indeed, through improved educational efforts and accessibility, patients are increasingly informed of MHV-specific risks of bleeding/endocarditis. While this is mitigated with lifelong anticoagulation, anticoagulation itself poses significant deadly risks and requires significant lifestyle changes (4). Some patients may prefer BHVs because they weigh quality of life over long-term survival (4). The advent of ViV procedures, among other minimally invasive cardiac techniques, may imminently permit bioprosthetic reinsertion at reintervention. These factors likely drive the decreasing age cut-off for BHVs. The bioroot Bentall is a promising alternative procedure, driven by a shifting demographic of surgical candidates: an aging population with increasing life expectancy. In addition to increased aortic root disease with increasing age, younger patients are requesting solutions which circumvent the lifelong burden of anticoagulation. Nevertheless, access to composite preassembled grafts remains region-dependent. Variability in graft selection, surgical expertise, and healthcare infrastructure affects widespread adoption worldwide.

## 4 Conclusion

### 4.1 Future perspectives and knowledge gaps

We believe that key avenues of innovation should focus on reducing reoperation rates. Technological advances should seek to increase BHV durability, developing improved calcification resistance, structural integrity, and bioengineered tissue scaffolds, offering patients the advantages of bioprosthetics without traditional pitfalls. Given the success of ViV to address BHV degeneration, another research priority should be to minimize IE, a complication necessitating complete root rereplacement (47). Strategies may involve infection-resistant biomaterials, enhanced antimicrobial coatings, and novel immune-modulating therapies.

### 4.2 Modern paradigm

Skirted, supra-annular BHV seating enlarges EOA in small annuli, reduces PPM risk, and preserves favourable geometry for ViV/reintervention. Minimally-invasive and robotic-assisted Bentall procedures also open the door for enhanced recovery. For example, the use of Bentall with partial upper sternotomy, which is not associated with improved cross-clamp or perfusion times or overall morality, may reduce postoperative pain and length of stay (48). Moreover, bioengineered graft materials remain an exciting area of research. As advancements progress, the bioroot Bentall technique continues to evolve, bridging the gap between mechanical durability and bioprosthetic adaptability, ultimately adapting to the modern world of aorto-valvular surgery.
